# Case Report: Prenatal diagnosis of novel compound heterozygous variants in *WDR35* gene causing short-rib thoracic dysplasia 7 with or without polydactyly

**DOI:** 10.3389/fped.2024.1503455

**Published:** 2025-01-14

**Authors:** Jianlong Zhuang, Junyu Wang, Zhengping Huang, Yu’e Chen, Chunnuan Chen

**Affiliations:** ^1^ Prenatal Diagnosis Center, Quanzhou Women’s and Children’s Hospital, Quanzhou, China; ^2^ Department of Neurology, Second Affiliated Hospital of Fujian Medical University, Quanzhou, China; ^3^ Department of Ultrasound, Quanzhou Women’s and Children’s Hospital, Quanzhou, China

**Keywords:** whole exome sequencing, chromosomal microarray analysis, *WDR35*, *NPHP4*, etiology diagnosis

## Abstract

**Background:**

Whole exome sequencing (WES) technology has been increasingly used for the etiological diagnosis of fetuses with ultrasound anomalies. In this article, we report a novel deletion compound combined with a causative variant in *WDR35* gene leading to short-rib thoracic dysplasia 7 (SRTD7) with or without polydactyly using WES.

**Methods:**

This study involved a Chinese fetus with clinical features of skeletal dysplasia on ultrasound imaging, in whom chromosome abnormalities and copy number variants (CNVs) were detected by chromosomal microarray analysis (CMA), and sequence variants were detected by WES. The obtained results were further verified by Sanger sequencing or real time quantitative PCR (qPCR).

**Results:**

No chromosomal abnormality or CNVs were identified in the fetus by CMA. However, WES result revealed a 14.38-kb large novel deletion compound covering exon 7 to exon 12 combined with a missense variant NM_001006657.2:c.932G>T(p.W311l) in *WDR35*. Both variants were thought of as pathogenic, which was further confirmed by Sanger sequencing and qPCR. In addition, two compound heterozygous variants NM_015102.5:c.[1196A>G(p.E399G)];[1972C>T(p.R658*)] in *NPHP4* gene were also identified in the fetus, which may be partially responsible for fetal kidney hyperechogenicity and oligohydramnios.

**Conclusion:**

This is the first study reporting a novel deletion compound combined with a causative missense variant in *WDR35* leading to SRTD7. This finding may broaden the spectrum of variants of *WDR35* gene and provide a valuable reference for clinical counseling of related abnormalities in pregnancies.

## Introduction

Short-rib thoracic dysplasia 7 (SRTD7) with or without polydactyly is a group of autosomal recessive skeletal ciliopathies with short ribs and thoracic stenosis as the main phenotypes. SRTD7 with or without polydactyly is caused by homozygous or compound heterozygous mutations in *WDR35* gene. *WDR35* (OMM 613602), a WD40 domain-containing protein encoding 28 exons, is located on chromosome 2q24.1, playing an important role in intraflagellar transport (1). It is typically characterized by a constricted thoracic cage, short ribs, shortened tubular bones, and a “trident” appearance of the acetabular roof, other organ malformations, such as renal cysts, which phenotypically overlap with cranioectodermal dysplasias (CED), an autosomal recessive disorder characterized by sagittal craniosynostosis, dolichocephaly, ectodermal abnormalities, skeletal dysplasia, characteristic facial features, and other clinical anomalies (2, 3).

*WDR35* gene may also lead to CED. A previous study reported that SRTD7 affected the same organs as did CED with much greater severity, probably due to more severe mutations in *WDR* gene, such as deletion, non-sense mutations, or frameshift mutations (4). In this study, we report a 14.38-kb novel deletion compound combined with a causative variant in *WDR35* gene leading to SRTD7 in a Chinese family. In addition, we also identified two compounded heterozygous variants in *NPHP4* gene in the fetus of the said family, which may be partially responsible for fetal kidney hyperechogenicity and oligohydramnios.

## Case presentation

**Figure 1:**
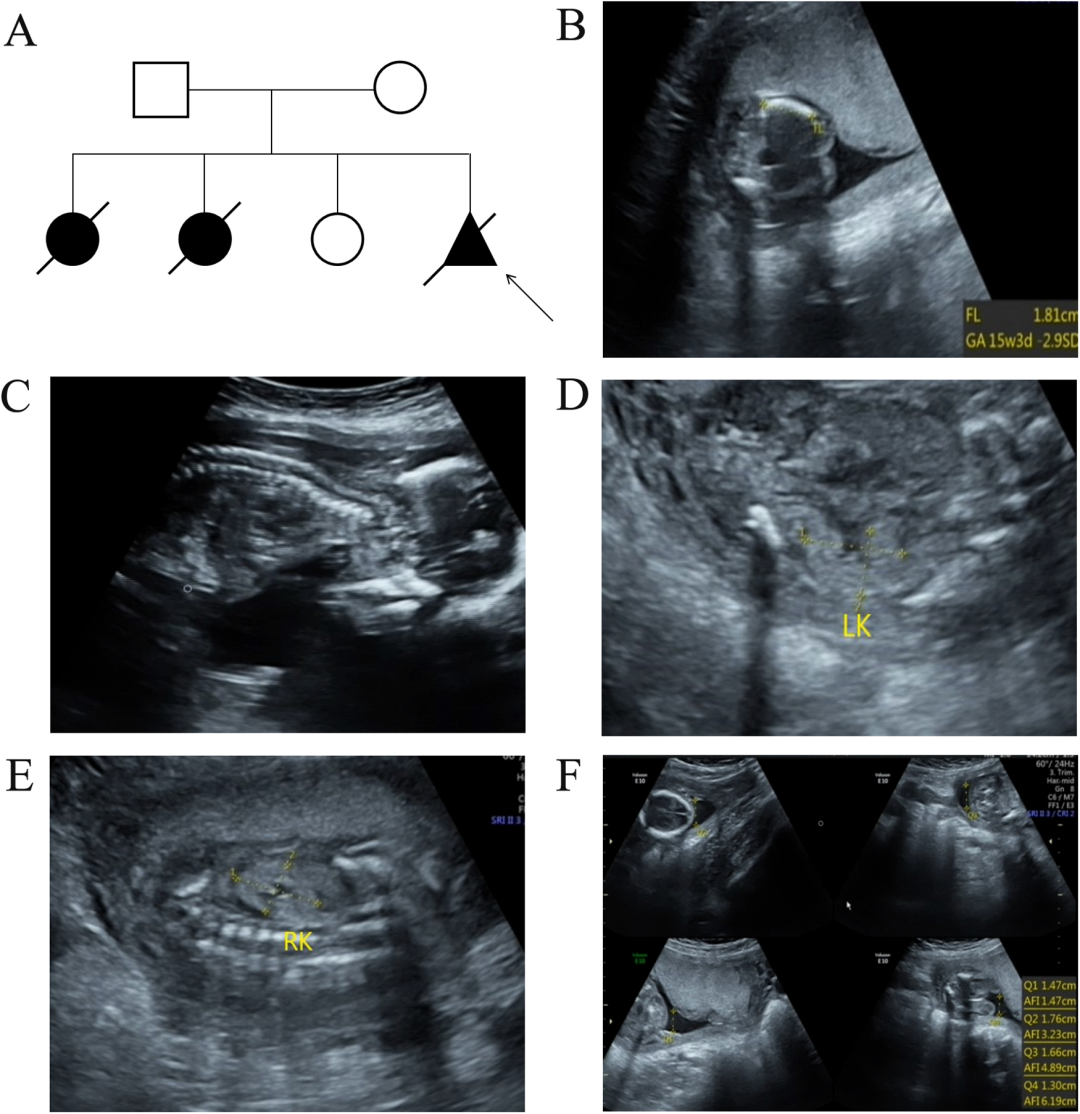
The pedigree information of the family and ultrasound examination results in the present fetus. **(A)** The pedigree analysis results of the enrolled family. The arrow indicates the proband. **(B)** Ultrasound examination results revealed short limbs and curved bones. **(C)** A narrow chest was also observed in the fetus. In addition, enhanced renal echogenicity **(D,E)** and oligohydramnios **(F)** was observed in the present fetus.

A 30-year-old G4P1 pregnant woman from Quanzhou, Southeast China, came to our hospital for genetic and etiological diagnosis at the gestational age of 18 weeks because of prenatal ultrasound anomalies. The couple denied consanguinity marriage and family-inherited diseases. Her first pregnancy was terminated because of short-limb deformities and congenital heart defects detected at the gestational age of 28 weeks. During her second pregnancy, similar ultrasound anomalies were observed and they chose to terminate the pregnancy. At her third pregnancy, no remarkable ultrasound anomalies were observed, and a full-term female infant was born who displayed normal developmental milestones at 5 years of age. This is her fourth pregnancy and several ultrasound anomalies were detected including short limbs, curved bones, narrow chest, enhanced renal echogenicity, oligohydramnios, enhanced intestinal echogenicity, and choroid plexus cyst (Figure 1). Finally, the family chose to terminate the pregnancy, and the aborted tissue was collected. After informed consent was signed, chromosomal microarray analysis (CMA) and whole exome sequencing (WES) were carried out to detect fetal copy number variants (CNVs) and sequence variants. No chromosomal abnormality was detected in the fetus by chromosomal microarray analysis.

**Figure 2:**
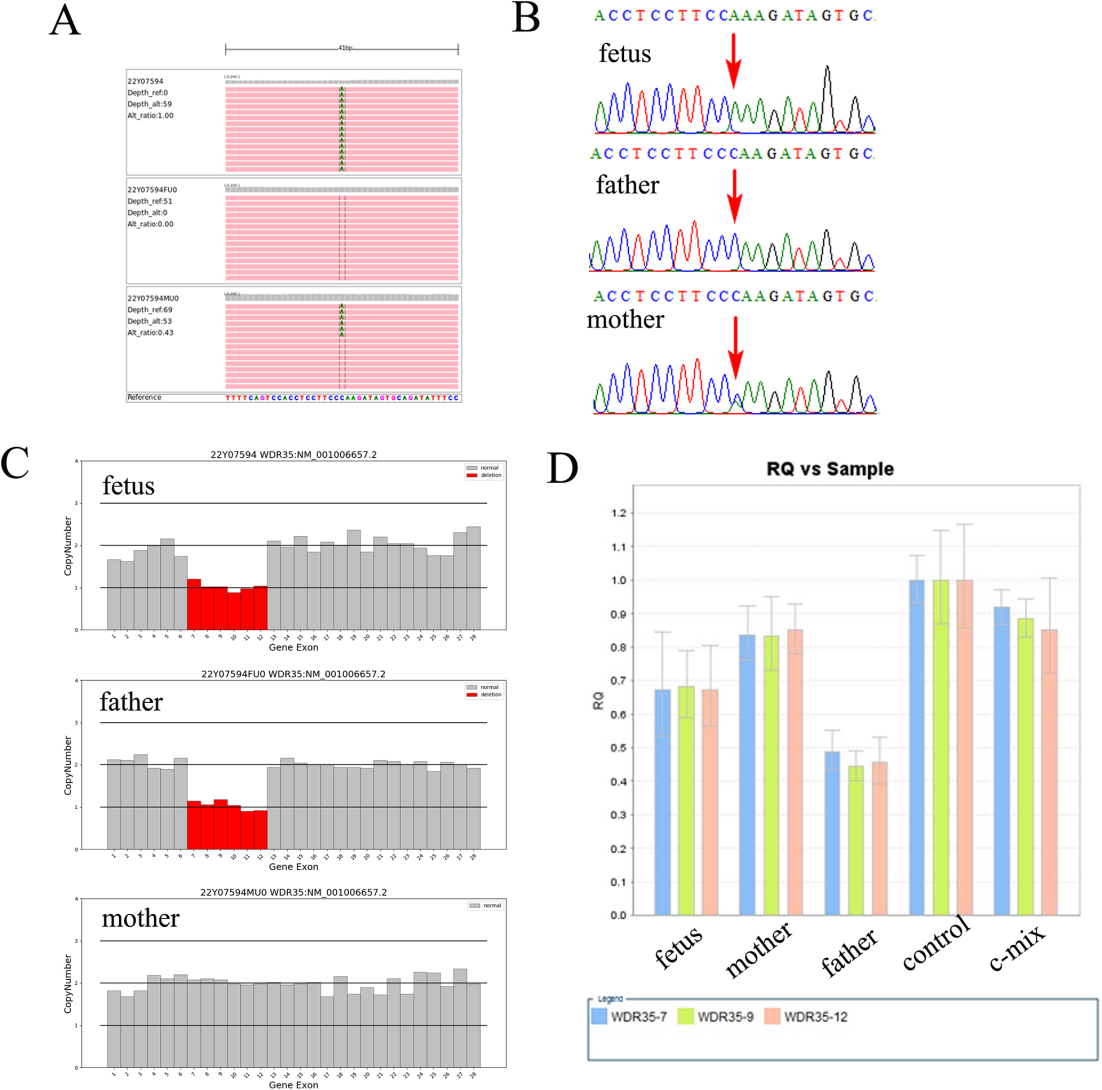
A 14.38-kb novel deletion compound with a missense variant in *WDR35* is identified in the fetus. **(A)** Trio-whole exome sequencing (trio-WES) demonstrated a missense variant NM_001006657.2:c.932G>T(p.W311l) in *WDR35* in the fetus. **(B)** Sanger sequencing verified the c.932G>T in the fetus, which was also present in the mother. **(C)** A 14.38-kb deletion compound encompassing exon 7 to exon 12 was also identified in the fetus using trio-WES. **(D)** qPCR analysis revealed a heterozygous deletion from exon 7 to exon 12 in *WDR35* in both the fetus and the father, but the fetus's mother did not have the deletion.

**Figure 3:**
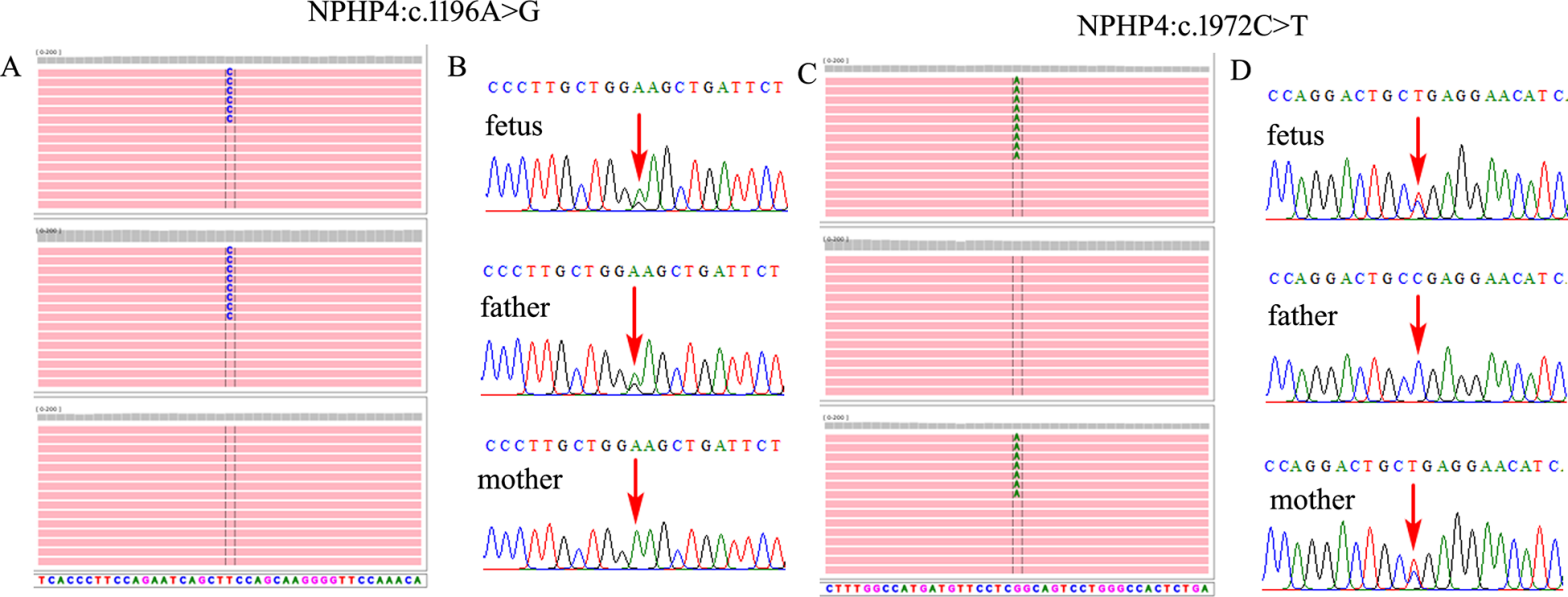
Two compounded heterozygous variants in *NPHP4* gene were identified in the fetus. **(A,C)** Trio-WES demonstrated an NM_015102.5:c.1196A>G(p.E399G) compounded with NM_015102.5:c.1972C>T(p.R658*) in *NPHP4* gene in the fetus, which was inherited from the parents, respectively. **(B,D)** Sanger sequencing verified both of the variants in the family.

Further WES was carried out to investigate relevant sequence variants in the affected fetus. The result revealed a 14.38-kb novel deletion compound covering exon 7 to exon 12, which was combined with a missense variant NM_001006657.2:c.932G>T(p.W311l) in *WDR35* in the fetus, which was believed to have been transmitted from the parent. According to the American College of Medical Genetics and Genomics (ACMG) guidelines, both variants were supposed to be pathogenic, which was further confirmed by Sanger sequencing and real time quantitative PCR (qPCR) (Figure 2). In addition, two compound heterozygous variants NM_015102.5:c.[1196A>G(p.E399G)];[1972C>T(p.R658*)] in *NPHP4* gene were also identified in the fetus, which were inherited from the parents, respectively. Both variants were further verified by Sanger sequencing (Figure 3). According to the ACMG guidelines, the variant of NM_015102.5:c.1972C>T(p.R658*) was classified as a pathogenic variant (PVS1 + PP1_Strong + PM2_Supporting + PM3_Supporting), and c.1196A>G(p.E399G) was interpreted as a variant of unknown significance. As predicted by Spidex software, the variant of c.1196A>G(p.E399G) may affect splicing (PP3).

## Discussion and conclusion

WES has become an increasingly popular technique for prenatal etiological diagnosis of genetic causes of fetal structural abnormalities including multiple fetal malformations, skeletal dysplasia, central nervous system (CNS) abnormalities, cardiovascular malformations, and other structural anomalies (5, 6). Previous research has shown that WES can offer a high positive detection rate for skeletal dysplasia (7, 8). In addition, a most recent study conducted by Zeng et al. (9) indicated that clinical exome sequencing exhibits an obvious advantage in detecting small CNVs over CMA. Skeletal ciliopathy is a rare disease characterized by abnormalities in the skeletal system, caused by genetic defects that regulate the structure or function of primary cilia. In the present study, we identified a large novel deletion compound with a causative variant in *WDR35* gene causing SRTD7 in a fetus with skeletal dysplasia, and two compound heterozygous variants in *NPHP4* gene, which may be partially responsible for fetal kidney hyperechogenicity and oligohydramnios.

High genetic heterogeneity is present in SRTD. SRTD7 was first described by Kannu et al. (10) in a New Zealand family classified as an “unclassifiable” short-rib polydactyly syndrome. Caparrós-Martín et al. (11) studied five patients from three unrelated families with short ribs, mesomelic shortening of limbs, and tooth and nail dysplasia, and homozygosity splice mutations in *WDR35* gene were identified. In addition, a previous study conducted by Duran et al. (12) reported three siblings and an unrelated female infant with compound heterozygosity mutations in *WDR35* gene leading to SRTD7. All three sibs exhibited short ribs, short limbs, bilateral postaxial polydactyly of the hands and feet with aphalangia of the hands, and bending of humeri, radii, and ulnae. However, the unrelated female infant did not have polydactyly. The fetus in the present study was found to have short limbs, curved bones, and narrow chest on prenatal ultrasound imaging, all of which were consistent with the clinical features of SRTD7, but no polydactyly was detected in this fetus. CED, which is also caused by WDR35 mutations but with milder phenotypes, is mainly characterized by craniosynostosis and ectodermal abnormalities. In addition, most *WDR35* gene variants leading to CED are missense mutations (4, 13, 14). By contrast, at least one allele in WDR35 gene had a loss-of-function in patients with SRTD7 (11, 12, 15). In the present study, a previous reported causative missense variant (c.932G>T) (16) compound with a large novel deletion in *WDR35* gene was identified, which provides more evidence the cause of SRTD7. In addition, this family also experienced two pregnancies with fetal short limbs, which may be due to compound heterozygous mutations in *WDR35* gene, but there is a lack of further genetic analysis.

In the present study, we also identified two compound heterozygous variants NM_015102.5:c.[1196A>G(p.E399G)];[1972C>T(p.R658*)] in *NPHP4* gene in the fetus. Nephronophthisis is an autosomal recessive kidney disease characterized by the multicystic dysplastic kidney, oligohydramnios, and tubulointerstitial nephritis that progresses to end-stage renal disease (17). At present, four genes (*NPHP1*, *NPHP2*, *NPHP3*, and *NPHP4*) have been identified as being responsible for nephronophthisis. *NPHP4* gene encoding nephrocystin-4 is known to cause end-stage renal disease in children and young adults (18). The pathogenic variant of c.1972C>T(p.R658*) has been identified in patients with nephronophthisis 4 and as being co-segregated with the disease in the family (19, 20). However, the c.1196A>G(p.E399G) variant was interpreted as an unclassified variant, but we cannot rule out that the detected *NPHP4* variants may partially be responsible for fetal kidney hyperechogenicity and oligohydramnios.

The variants identified in *WDR35* and *NPHP4* of the fetus in this study may be responsible for the ultrasound anomalies identified in the second trimester of pregnancy. However, further functional analysis is required to explore the pathogenicity of the c.1196A>G(p.E399G) variant in *NPHP4*. In addition, the specimen and specific clinical phenotypes of the previous two fetuses in this pregnant woman that had similar clinical abnormalities were not available in this study.

In this study, we presented a novel heterozygous compound variant in *WDR35* gene causing SRTD7 in a Chinese family, which may provide more insights into understanding the genotype and phenotype correlation. In addition, we also identified two compounded heterozygous variants in *NPHP4* gene in the fetus, which may be partially responsible for fetal kidney hyperechogenicity and oligohydramnios.

## Data Availability

The raw data supporting the conclusions of this article will be made available by the authors, without undue reservation.
